# *catena*-Poly[(diaqua­cadmium)-μ-4,4′-[sulfonyl­bis­(1,4-phenyl­ene­oxy)]­di­acet­ato-κ^4^ *O*,*O*′:*O*′′,*O*′′′]

**DOI:** 10.1107/S160053681200445X

**Published:** 2012-02-10

**Authors:** Zhan-Ling Ma

**Affiliations:** a College of Chemistry and Chemical Engineering of Bohai University, Jinzhou, Liaoning 121000, People’s Republic of China

## Abstract

In the title coordination polymer, [Cd(C_16_H_12_O_8_S)(H_2_O)_2_]_*n*_, the Cd^II^ ion is situated on a crystallographic twofold rotation axis, being coordinated by four O atoms from two bidentate 4,4′-[sulfonyl­bis­(1,4-phenyl­ene­oxy)]diacetate (*L*) ligands and two water mol­ecules in a highly distorted CdO_6_ octa­hedral geometry. Each complete ligand *L*, which is also generated by twofold symmetry with the S atom lying on the rotation axis, bridges two Cd^II^ atoms to form a polymeric zigzag chain propagating in the [10-1] direction. O—H⋯O hydrogen bonds between the coordinated water mol­ecules and carboxyl­ate O atoms are involved in the packing.

## Related literature

For related coordination polymers, see: Tanaka *et al.* (2008 ▶); Zheng *et al.* (2009 ▶, 2010 ▶).
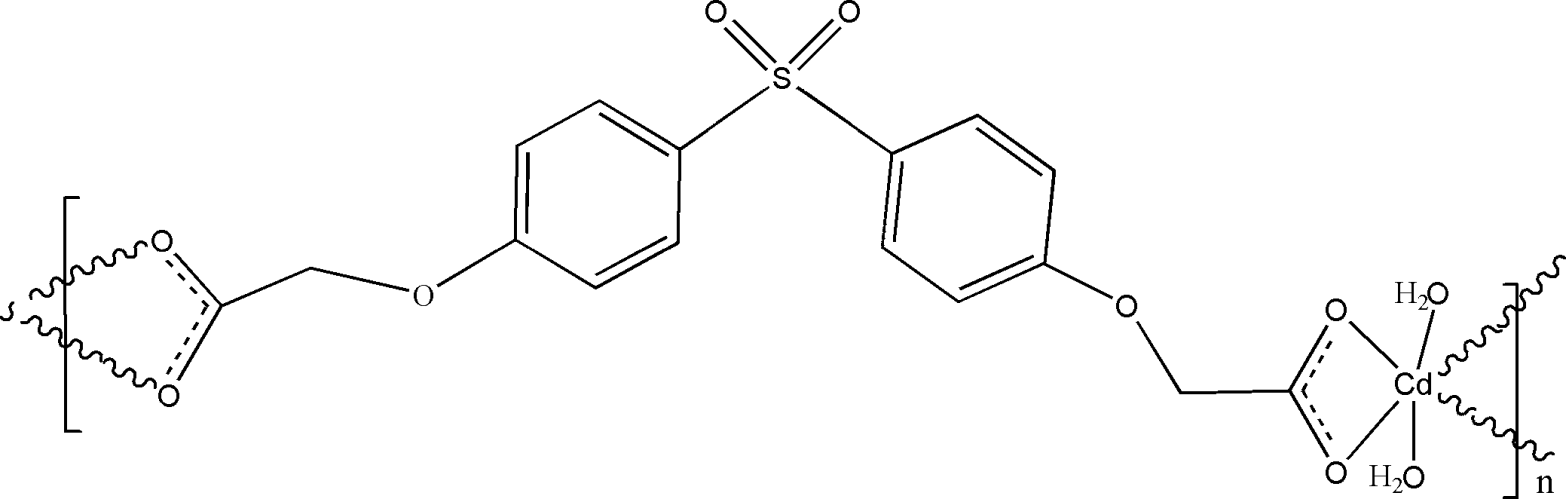


## Experimental

### 

#### Crystal data

[Cd(C_16_H_12_O_8_S)(H_2_O)_2_]
*M* *_r_* = 512.75
Monoclinic, 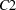

*a* = 11.9274 (11) Å
*b* = 5.3995 (5) Å
*c* = 14.8194 (14) Å
β = 111.692 (1)°
*V* = 886.81 (14) Å^3^
*Z* = 2
Mo *K*α radiation
μ = 1.41 mm^−1^
*T* = 295 K
0.15 × 0.14 × 0.12 mm

#### Data collection

Bruker APEXII CCD diffractometer
Absorption correction: multi-scan (*SADABS*; Bruker, 2005 ▶) *T*_min_ = 0.817, *T*_max_ = 0.850
2364 measured reflections
1240 independent reflections
1220 reflections with *I* > 2σ(*I*)
*R*_int_ = 0.020

#### Refinement

*R*[*F* ^2^ > 2σ(*F* ^2^)] = 0.020
*wR*(*F* ^2^) = 0.044
*S* = 1.00
1240 reflections
128 parameters
1 restraint
H-atom parameters constrained
Δρ_max_ = 0.23 e Å^−3^
Δρ_min_ = −0.34 e Å^−3^
Absolute structure: Flack (1983 ▶), 361 Friedel pairs
Flack parameter: 0.04 (3)

### 

Data collection: *APEX2* (Bruker, 2005 ▶); cell refinement: *SAINT* (Bruker, 2005 ▶); data reduction: *SAINT*; program(s) used to solve structure: *SHELXS97* (Sheldrick, 2008 ▶); program(s) used to refine structure: *SHELXL97* (Sheldrick, 2008 ▶); molecular graphics: *SHELXTL* (Sheldrick, 2008 ▶); software used to prepare material for publication: *SHELXTL*.

## Supplementary Material

Crystal structure: contains datablock(s) global, I. DOI: 10.1107/S160053681200445X/hb6572sup1.cif

Structure factors: contains datablock(s) I. DOI: 10.1107/S160053681200445X/hb6572Isup2.hkl

Additional supplementary materials:  crystallographic information; 3D view; checkCIF report

## Figures and Tables

**Table:** 

Cd1—O4	2.183 (3)
Cd1—O2	2.323 (3)
Cd1—O3	2.405 (2)

**Table:** 

O2—Cd1—O3	55.32 (8)

**Table 2:** Hydrogen-bond geometry (Å, °)

*D*—H⋯*A*	*D*—H	H⋯*A*	*D*⋯*A*	*D*—H⋯*A*
O4—H9⋯O2^i^	0.85	1.87	2.691 (4)	163
O4—H10⋯O3^ii^	0.85	1.87	2.699 (3)	164

Symmetry codes: (i) 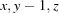; (ii) 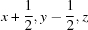.

**Fig. 1.**
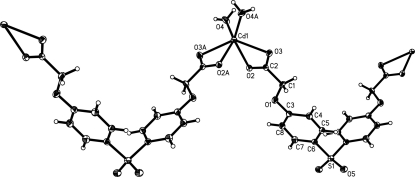
Crystal structure of the title compound with labeling and 30% probability displacement ellopsoids (H atoms are shown as spheres of arbitrary size) [Symmetry codes: (A) -x + 2, y, -z + 1].

**Fig. 2.**
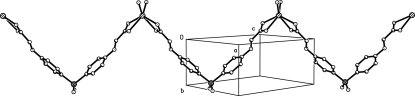
The one-dimensional zigzag chain structure of the title compound.

## supplementary crystallographic information

### Comment

A large family of coordination polymers has been developed recently owing to their potential applications as functional solid materials and their intriguing architectures or topologies. In the past ten years, there has been a growing interest in metal-organic frameworks involving semi-rigid V-shaped dicarboxylate ligands (Tanaka *et al.*, 2008; Zheng *et al.*, 2009, 2010). 4,4'-sulfonyldi-*p*-phenylenedioxydiacetic acid is a typical example of a semi-rigid V-shaped dicarboxylate ligand. To the best of our knowledge, there has been no report about its coordination compounds. Recently, we obtained the title cadium polymer (I), its crystal structure is reported here.

In the structure of (I) each cadium(II) atom is coordinated by four oxygen atoms from two 4,4'-sulfonyldi-*p*-phenylenedioxydiacetate ligands and two water molecules, displays a highly distorted octahedral geometry(Fig. 1). Each ligand *L* bridges two cadium(II) centers to form polymeric zigzag chain propagated in direction [101](Fig. 2). Moreover, there are Intermolecular O—H···O hydrogen bonds between the coordinated water molecules and the carboxylate O atoms consolidate the further crystal packing(Table 1).

### Experimental

A mixture of Cd(Ac)_2_(0.5 mmol), 4,4'-sulfonyldi-*p*- phenylenedioxydiacetic acid (0.5 mmol), NaOH (1 mmol) and H_2_O (15 ml) was placed in a 23 ml Teflon reactor, which was heated at 413 K for three days and then cooled to room temperature. Colourless blocks were obtained on cooling, which were washed with water and dried in air.

### Refinement

All H atoms were placed in idealized positions (O—H = 0.85 Å and C—H = 0.93–0.97 Å) and refined as riding atoms with *U*_iso_(H) = 1.2*U*_eq_(C) and *U*_iso_(H) = 1.5*U*_eq_(O).

### Crystal data

**Table:**

[Cd(C_16_H_12_O_8_S)(H_2_O)_2_]	*F*(000) = 512
*M**_r_* = 512.75	*D*_x_ = 1.920 Mg m^−^^3^
Monoclinic, *C*2	Mo *K*α radiation, λ = 0.71073 Å
Hall symbol: C 2y	Cell parameters from 1449 reflections
*a* = 11.9274 (11) Å	θ = 3.0–26.0°
*b* = 5.3995 (5) Å	µ = 1.41 mm^−^^1^
*c* = 14.8194 (14) Å	*T* = 295 K
β = 111.692 (1)°	Block, colorless
*V* = 886.81 (14) Å^3^	0.15 × 0.14 × 0.12 mm
*Z* = 2	

### Data collection

**Table:**

Bruker APEXII CCD diffractometer	1240 independent reflections
Radiation source: fine-focus sealed tube	1220 reflections with *I* > 2σ(*I*)
Graphite monochromator	*R*_int_ = 0.020
φ and ω scans	θ_max_ = 25.1°, θ_min_ = 1.5°
Absorption correction: multi-scan (*SADABS*; Bruker, 2005)	*h* = −14→12
*T*_min_ = 0.817, *T*_max_ = 0.850	*k* = −3→6
2364 measured reflections	*l* = −17→16

### Refinement

**Table:**

Refinement on *F*^2^	Secondary atom site location: difference Fourier map
Least-squares matrix: full	Hydrogen site location: inferred from neighbouring sites
*R*[*F*^2^ > 2σ(*F*^2^)] = 0.020	H-atom parameters constrained
*wR*(*F*^2^) = 0.044	*w* = 1/[σ^2^(*F*_o_^2^) + (0.0235*P*)^2^ + 0.0192*P*] where *P* = (*F*_o_^2^ + 2*F*_c_^2^)/3
*S* = 1.00	(Δ/σ)_max_ < 0.001
1240 reflections	Δρ_max_ = 0.23 e Å^−^^3^
128 parameters	Δρ_min_ = −0.34 e Å^−^^3^
1 restraint	Absolute structure: Flack (1983), 361 Friedel pairs
Primary atom site location: structure-invariant direct methods	Flack parameter: 0.04 (3)

### Special details

**Table:**

Geometry. All e.s.d.'s (except the e.s.d. in the dihedral angle between two l.s. planes) are estimated using the full covariance matrix. The cell e.s.d.'s are taken into account individually in the estimation of e.s.d.'s in distances, angles and torsion angles; correlations between e.s.d.'s in cell parameters are only used when they are defined by crystal symmetry. An approximate (isotropic) treatment of cell e.s.d.'s is used for estimating e.s.d.'s involving l.s. planes.
Refinement. Refinement of *F*^2^ against ALL reflections. The weighted *R*-factor *wR* and goodness of fit *S* are based on *F*^2^, conventional *R*-factors *R* are based on *F*, with *F* set to zero for negative *F*^2^. The threshold expression of *F*^2^ > σ(*F*^2^) is used only for calculating *R*-factors(gt) *etc*. and is not relevant to the choice of reflections for refinement. *R*-factors based on *F*^2^ are statistically about twice as large as those based on *F*, and *R*- factors based on ALL data will be even larger.

### Fractional atomic coordinates and isotropic or equivalent isotropic displacement parameters (Å^2^)

**Table:**

	*x*	*y*	*z*	*U*_iso_*/*U*_eq_	
Cd1	1.0000	−0.5416	0.5000	0.02782 (11)	
S1	0.5000	0.8539 (2)	0.0000	0.0263 (3)	
O1	0.7967 (2)	0.1490 (5)	0.2934 (2)	0.0378 (7)	
O2	0.9342 (2)	−0.2296 (5)	0.38439 (17)	0.0291 (6)	
O3	0.7996 (2)	−0.3705 (5)	0.44023 (18)	0.0329 (6)	
O4	1.0681 (2)	−0.8139 (5)	0.4240 (2)	0.0464 (8)	
H10	1.1352	−0.8433	0.4183	0.070*	
H9	1.0227	−0.9369	0.3993	0.070*	
O5	0.4191 (2)	0.9887 (8)	0.03394 (16)	0.0355 (7)	
C1	0.7421 (3)	−0.0213 (13)	0.3363 (2)	0.0287 (8)	
H1A	0.6732	−0.0974	0.2865	0.034*	
H1B	0.7134	0.0647	0.3811	0.034*	
C2	0.8317 (3)	−0.2201 (7)	0.3903 (2)	0.0263 (8)	
C3	0.7219 (3)	0.3149 (7)	0.2293 (3)	0.0287 (9)	
C4	0.6016 (3)	0.3498 (7)	0.2148 (3)	0.0299 (8)	
H4	0.5662	0.2610	0.2510	0.036*	
C5	0.5347 (3)	0.5185 (6)	0.1457 (2)	0.0278 (11)	
H5	0.4537	0.5432	0.1353	0.033*	
C6	0.5877 (3)	0.6509 (7)	0.0921 (2)	0.0248 (8)	
C7	0.7089 (3)	0.6174 (7)	0.1081 (3)	0.0312 (9)	
H7	0.7450	0.7081	0.0728	0.037*	
C8	0.7754 (3)	0.4480 (16)	0.1770 (2)	0.0318 (8)	
H8	0.8567	0.4240	0.1880	0.038*	

### Atomic displacement parameters (Å^2^)

**Table:**

	*U*^11^	*U*^22^	*U*^33^	*U*^12^	*U*^13^	*U*^23^
Cd1	0.02314 (18)	0.02072 (17)	0.0399 (2)	0.000	0.01196 (14)	0.000
S1	0.0293 (7)	0.0238 (6)	0.0259 (7)	0.000	0.0104 (6)	0.000
O1	0.0261 (15)	0.0370 (16)	0.0488 (17)	0.0028 (12)	0.0121 (13)	0.0190 (14)
O2	0.0236 (14)	0.0289 (14)	0.0338 (14)	−0.0016 (11)	0.0093 (11)	0.0018 (12)
O3	0.0250 (14)	0.0341 (16)	0.0397 (15)	0.0016 (11)	0.0121 (12)	0.0111 (12)
O4	0.0321 (16)	0.0350 (17)	0.082 (2)	−0.0108 (13)	0.0322 (15)	−0.0233 (15)
O5	0.0391 (13)	0.031 (2)	0.0373 (12)	0.0036 (16)	0.0152 (10)	−0.0078 (15)
C1	0.0239 (16)	0.028 (2)	0.0323 (16)	0.008 (3)	0.0082 (13)	0.006 (3)
C2	0.022 (2)	0.0256 (19)	0.0265 (19)	−0.0037 (15)	0.0030 (16)	−0.0013 (16)
C3	0.027 (2)	0.027 (2)	0.0281 (19)	−0.0019 (16)	0.0057 (17)	0.0037 (16)
C4	0.029 (2)	0.033 (2)	0.029 (2)	−0.0018 (16)	0.0119 (18)	0.0063 (15)
C5	0.0245 (18)	0.028 (3)	0.0309 (18)	0.0018 (14)	0.0099 (15)	0.0016 (14)
C6	0.026 (2)	0.0250 (19)	0.0223 (18)	−0.0019 (15)	0.0073 (15)	−0.0029 (16)
C7	0.031 (2)	0.036 (2)	0.031 (2)	−0.0013 (17)	0.0156 (18)	0.0018 (17)
C8	0.0247 (16)	0.035 (2)	0.0379 (18)	−0.001 (3)	0.0145 (13)	0.005 (3)

### Geometric parameters (Å, º)

**Table:**

Cd1—O4^i^	2.183 (3)	O4—H9	0.8500
Cd1—O4	2.183 (3)	C1—C2	1.518 (6)
Cd1—O2^i^	2.323 (3)	C1—H1A	0.9700
Cd1—O2	2.323 (3)	C1—H1B	0.9700
Cd1—O3^i^	2.405 (2)	C3—C8	1.375 (6)
Cd1—O3	2.405 (2)	C3—C4	1.383 (5)
S1—O5	1.439 (3)	C4—C5	1.382 (5)
S1—O5^ii^	1.439 (3)	C4—H4	0.9300
S1—C6^ii^	1.762 (4)	C5—C6	1.383 (5)
S1—C6	1.762 (4)	C5—H5	0.9300
O1—C3	1.369 (5)	C6—C7	1.387 (5)
O1—C1	1.407 (6)	C7—C8	1.382 (7)
O2—C2	1.259 (4)	C7—H7	0.9300
O3—C2	1.249 (4)	C8—H8	0.9300
O4—H10	0.8499		
			
O4^i^—Cd1—O4	95.33 (15)	O1—C1—C2	110.3 (3)
O4^i^—Cd1—O2^i^	101.35 (10)	O1—C1—H1A	109.6
O4—Cd1—O2^i^	141.27 (9)	C2—C1—H1A	109.6
O4^i^—Cd1—O2	141.27 (9)	O1—C1—H1B	109.6
O4—Cd1—O2	101.35 (10)	C2—C1—H1B	109.6
O2^i^—Cd1—O2	87.01 (13)	H1A—C1—H1B	108.1
O4^i^—Cd1—O3^i^	125.27 (10)	O3—C2—O2	122.2 (3)
O4—Cd1—O3^i^	86.56 (9)	O3—C2—C1	117.5 (3)
O2^i^—Cd1—O3^i^	55.32 (8)	O2—C2—C1	120.3 (3)
O2—Cd1—O3^i^	90.67 (9)	O1—C3—C8	114.5 (4)
O4^i^—Cd1—O3	86.56 (9)	O1—C3—C4	124.7 (4)
O4—Cd1—O3	125.27 (10)	C8—C3—C4	120.8 (4)
O2^i^—Cd1—O3	90.67 (9)	C5—C4—C3	119.1 (3)
O2—Cd1—O3	55.32 (8)	C5—C4—H4	120.4
O3^i^—Cd1—O3	134.81 (13)	C3—C4—H4	120.4
O5—S1—O5^ii^	119.3 (4)	C4—C5—C6	120.3 (3)
O5—S1—C6^ii^	107.61 (17)	C4—C5—H5	119.8
O5^ii^—S1—C6^ii^	109.06 (16)	C6—C5—H5	119.8
O5—S1—C6	109.06 (16)	C5—C6—C7	120.2 (3)
O5^ii^—S1—C6	107.61 (17)	C5—C6—S1	120.0 (3)
C6^ii^—S1—C6	103.0 (2)	C7—C6—S1	119.8 (3)
C3—O1—C1	116.7 (3)	C8—C7—C6	119.3 (3)
C2—O2—Cd1	92.9 (2)	C8—C7—H7	120.3
C2—O3—Cd1	89.3 (2)	C6—C7—H7	120.3
Cd1—O4—H10	136.1	C3—C8—C7	120.2 (3)
Cd1—O4—H9	116.7	C3—C8—H8	119.9
H10—O4—H9	106.5	C7—C8—H8	119.9
			
O4^i^—Cd1—O2—C2	14.8 (3)	C1—O1—C3—C8	−169.3 (4)
O4—Cd1—O2—C2	128.5 (2)	C1—O1—C3—C4	9.8 (6)
O2^i^—Cd1—O2—C2	−89.6 (2)	O1—C3—C4—C5	−178.2 (3)
O3^i^—Cd1—O2—C2	−144.9 (2)	C8—C3—C4—C5	0.9 (6)
O3—Cd1—O2—C2	3.3 (2)	C3—C4—C5—C6	−0.1 (5)
C2^i^—Cd1—O2—C2	−117.5 (2)	C4—C5—C6—C7	−0.8 (5)
O4^i^—Cd1—O3—C2	−176.1 (2)	C4—C5—C6—S1	176.8 (3)
O4—Cd1—O3—C2	−82.0 (2)	O5—S1—C6—C5	41.8 (3)
O2^i^—Cd1—O3—C2	82.6 (2)	O5^ii^—S1—C6—C5	172.5 (3)
O2—Cd1—O3—C2	−3.3 (2)	C6^ii^—S1—C6—C5	−72.3 (3)
O3^i^—Cd1—O3—C2	44.85 (19)	O5—S1—C6—C7	−140.6 (3)
C2^i^—Cd1—O3—C2	65.7 (3)	O5^ii^—S1—C6—C7	−9.9 (4)
C3—O1—C1—C2	169.6 (3)	C6^ii^—S1—C6—C7	105.2 (3)
Cd1—O3—C2—O2	5.9 (3)	C5—C6—C7—C8	1.0 (6)
Cd1—O3—C2—C1	−173.9 (3)	S1—C6—C7—C8	−176.6 (4)
Cd1—O2—C2—O3	−6.1 (4)	O1—C3—C8—C7	178.4 (5)
Cd1—O2—C2—C1	173.7 (3)	C4—C3—C8—C7	−0.7 (8)
O1—C1—C2—O3	173.0 (3)	C6—C7—C8—C3	−0.2 (8)
O1—C1—C2—O2	−6.7 (5)		

Symmetry codes: (i) −*x*+2, *y*, −*z*+1; (ii) −*x*+1, *y*, −*z*.

### Hydrogen-bond geometry (Å, º)

**Table:**

*D*—H···*A*	*D*—H	H···*A*	*D*···*A*	*D*—H···*A*
O4—H9···O2^iii^	0.85	1.87	2.691 (4)	163
O4—H10···O3^iv^	0.85	1.87	2.699 (3)	164

Symmetry codes: (iii) *x*, *y*−1, *z*; (iv) *x*+1/2, *y*−1/2, *z*.
